# Natural lecithin promotes neural network complexity and activity

**DOI:** 10.1038/srep25777

**Published:** 2016-05-27

**Authors:** Shahrzad Latifi, Ali Tamayol, Rouhollah Habibey, Reza Sabzevari, Cyril Kahn, David Geny, Eftekhar Eftekharpour, Nasim Annabi, Axel Blau, Michel Linder, Elmira Arab-Tehrany

**Affiliations:** 1 Neuroscience and Brain Technologies Department, Istituto Italiano di Tecnologia, via Morego 30, 16163 Genova, Italy; 2 Department of Neurology, David Geffen School of Medicine, University of California Los Angeles, Los Angeles, California, USA; 3 Regenerative Medicine Program, University of Manitoba, Winnipeg, MB, Canada; 4 Department of Medicine, Brigham and Women’s Hospital, Harvard Medical School, Boston, MA 02139, USA; 5 Robotics and Perception Group, Artificial Intelligence Lab, University of Zurich, Andreasstrasse 15, 8050 Zurich, Switzerland; 6 IFSTTAR, LBA, F-13015, Marseille, France; 7 Imaging Center for Psychiatry and Neurosciences, INSERM U894, Paris, France; 8 Department of Chemical Engineering, Northeastern University, 360 Huntington Avenue, Boston, MA 02115; 9 Université de Lorraine, Laboratoire d’Ingénierie des Biomolécules, 2, Avenue de la Forêt de Haye, F-54504 Vandoeuvre-lès-Nancy Cedex, France

## Abstract

Phospholipids in the brain cell membranes contain different polyunsaturated fatty acids (PUFAs), which are critical to nervous system function and structure. In particular, brain function critically depends on the uptake of the so-called “essential” fatty acids such as omega-3 (n-3) and omega-6 (n-6) PUFAs that cannot be readily synthesized by the human body. We extracted natural lecithin rich in various PUFAs from a marine source and transformed it into nanoliposomes. These nanoliposomes increased neurite outgrowth, network complexity and neural activity of cortical rat neurons *in vitro*. We also observed an upregulation of synapsin I (SYN1), which supports the positive role of lecithin in synaptogenesis, synaptic development and maturation. These findings suggest that lecithin nanoliposomes enhance neuronal development, which may have an impact on devising new lecithin delivery strategies for therapeutic applications.

Lecithin is a mixture of neutral lipids and phospholipids, which are significant constituents of the central nervous system (CNS). The phospholipids in the brain’s cell membranes contain many different fatty acids, of which omega-3 polyunsaturated fatty acid (PUFA) and docosahexaenoic acid (DHA) are the most abundant^1^. DHA and omega-3 are particularly important for normal brain development and cognitive function^2^,^3^,^4^,^5^. Animal-derived phospholipids usually contain phosphatidylethanolamine (PE), phosphatidylcholine (PC), phosphatidylserine (PS), phosphatidylinositol (PI), sphingomyelin and other glycerol phospholipids with complex fatty acid compositions. Numerous studies have demonstrated that PUFAs of the n-3 series including eicosapentaenoic acid (EPA, 20:5n-3) and DHA (22:6n-3) play key roles in a number of physiological processes^6^,^7^. Consequently, a strong link exists between an adequate supply of dietary PUFAs and the sustenance of cognitive health, learning, neural plasticity, synaptogenesis and synaptic transmission^8^,^9^,^10^,^11^. The phosphatides PC, PE, PS and PI are abundant in synaptic membranes and are involved in several fundamental steps of neurotransmission, including synaptic vesicle (SV) fusion and retrieval. In addition, lipid messengers are continuously produced during cerebral metabolic activity, which can directly modulate synaptic strength. It has been shown that dendritic arborization and stabilization are closely correlated to the strength and activity of synaptic inputs received by developing neurons. In addition, during the maturation of neurons and their synapses, synaptic activity plays a fundamental role in regulating dendritic stability and arbor development^12^. Moreover, it is well documented that more synaptic connections can be made with higher numbers of dendritic and axonal branches^12^. Therefore, the factors that control dendritic architecture and manipulate neurite outgrowth could potentially alter neural function and circuit properties by affecting synaptic compartments^13^.

Several studies have demonstrated the positive effect of PUFAs on brain development and function. For example, it has been shown that oral DHA administration promotes the synthesis of synaptic membranes, elevating the level of phosphatides and specific pre- and post-synaptic proteins^14^. Moreover, other studies have shown that DHA and EPA can increase the number of dendritic spines and potentially synapses on hippocampal neurons, particularly on excitatory glutamatergic synapses^15^. Darios and Davletov demonstrated that specific omega-3 and omega-6 PUFAs stimulated neurite outgrowth^4^.

Despite several studies on the role of PUFAs in brain function, there is no naturally-derived material with a PUFA composition similar to those found in the CNS. Moreover, the effect of the combinatorial use of essential PUFAs on neuronal function has not yet been well understood. In this work, we have developed a natural lecithin rich in 15 various fatty acids (saturated, mono and polyunsaturated) including DHA, EPA, linoleic, linolenic, and arachidonic acids with a high similarity to brain membrane components. We aim to study the effect of this biomimetic composition on the activity and function of primary cortical neurons. To improve the treatment efficiency, we engineered nanoliposomes with controlled size from the natural composition, herein called F22. Our *in vitro* data demonstrated the positive effect of F22 in stimulating neurite outgrowth, network formation and activity in neuronal cultures.

## Results and Discussions

**Figure 1:**
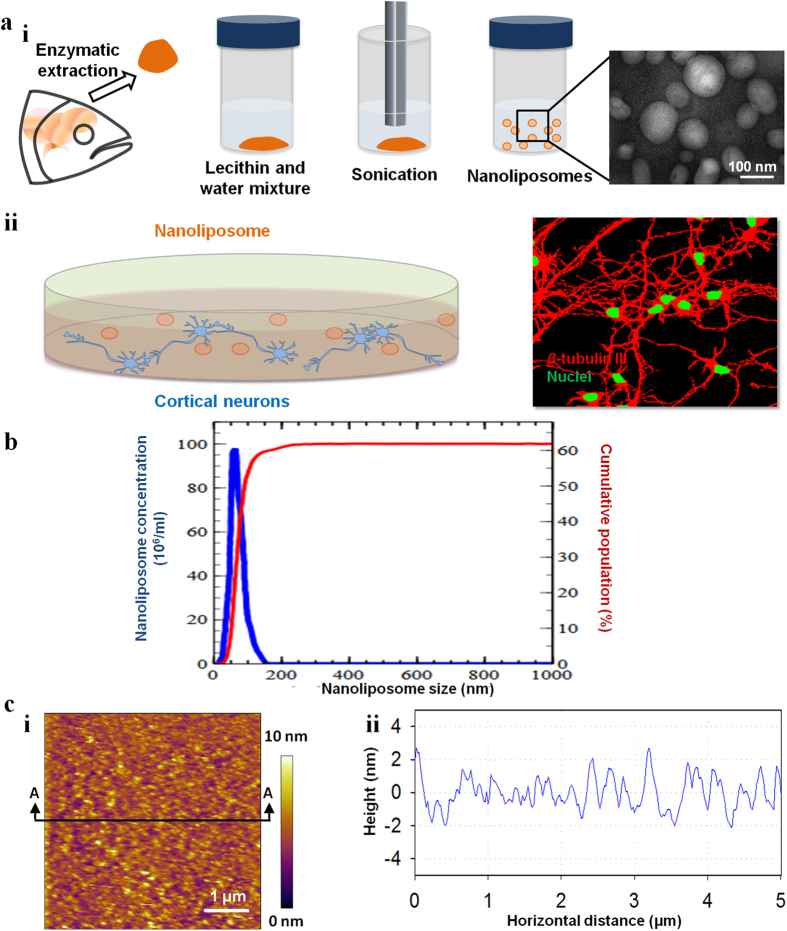
Formation and characterization of nanoliposomes from natural lecithin (F22). (**a**) Schematic of the study, (i) demonstrating the extraction of lecithin from a marine source followed by the generation of nanoliposomes; the inset shows a representative TEM image of the fabricated nanoliposomes. (ii) Culture of primary cortical neurons treated by nanoliposomes, which induced complex network formation in treated cultures at early age (right). (**b**) Size distribution of the fabricated nanoliposomes measured by NTA. (**c**) AFM images of a thin F22 layer (left) and its surface morphology along the A-A section (right).

The composition of F22, which was extracted from salmon heads through an enzymatic process^16^, mimics that of fatty acids and polar head phospholipids found in the human brain. We characterized the physico-chemical properties of the extracted lecithin and fabricated nanoliposomes. The separation of fatty acid esters (FAMEs) was carried out via gas chromatography. It was confirmed that 7 different PUFAs (>50% of the total fatty acid composition) were present in F22 (Table S1). The most significant PUFAs in F22 were C22:6 n-3 (DHA), C20:5n-3 (EPA), C18:2n-6 (linoleic acid) and C18:2n-3 (linolenic acid) with 30, 10, 6 and 5% content, respectively. C18:1n-9 (20%) was the most abundant monounsaturated fatty acid in the composition. The lipid composition of F22 was determined by thin-layer chromatography. The results indicated that phosphatidylcholine was the most abundant class of phospholipids in F22 (32% w/w). To facilitate and improve the uptake of F22, we generated nanoliposomes from the compound through emulsifying 2% lecithin in phosphate buffer saline (PBS) (Fig. 1a). The size of the generated nanoliposomes was determined using three different complementary techniques including nanoparticle tracking analysis (NTA), dynamic light scattering (DLS) and analysis of incoherent polarized steady light transport (AIPSLT). The advantage of the AIPLST technique over DLS and NTA is its ability to measure the particle size of turbid samples without any dilution (Fig. 1b, see Suppl. Info.). The results obtained from these three techniques showed similar size distribution. The nanoliposomes had a narrow size distribution with an average hydrodynamic diameter of 61 nm, a polydispersity index of 0.25, and an electrophoretic mobility of −5.11 μm.cm/(V.s) (Figs 1b and S2).

The morphological properties of nanoliposomes were determined by transmission electron microscopy (TEM) (Fig. 1a (i), see Supp. Info. for more details). To determine the homogeneity of the F22 lecithin, thin membranes were fabricated and atomic force microscopy (AFM) was carried out to image their topography (Fig. 1c, see Supp. Info. for more details). The results did not show any phase segregation.

**Figure 2:**
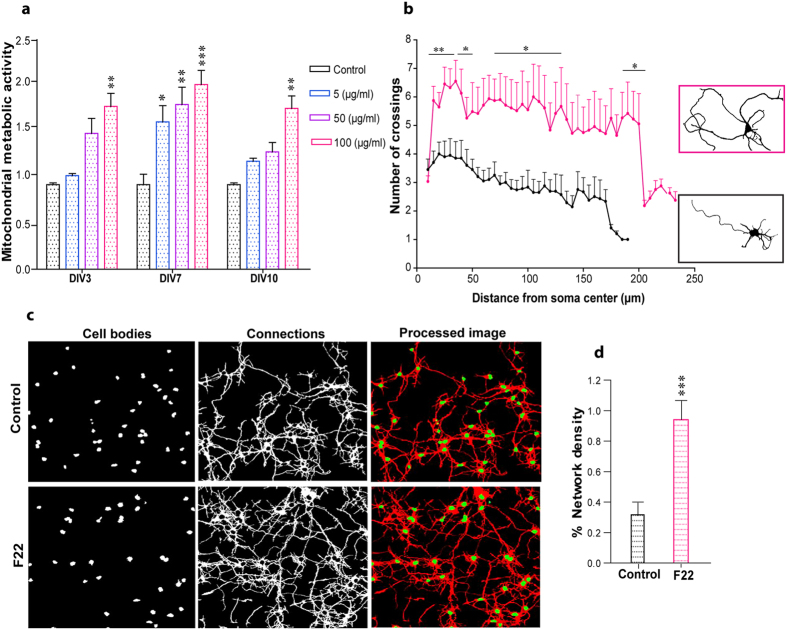
Effect of F22 on neuronal activity and network formation. (**a**) Mitochondrial metabolic activity of cortical neurons treated with different concentrations of F22 was measured at 3, 7 and 10 DIV. One-way ANOVA followed by the Bonferroni post-hoc test (*p < 0.05, **p < 0.01, ***p < 0.001; n = 17 samples from 3 independent experiments; * vs control). (**b**) Sholl analysis of cortical neurons treated with F22 at 100 μg/ml for 3 days compared to non-treated control cultures. Right: representative images of a single neuron under control conditions (black frame) and after treatment with F22 (pink frame). Two-way analysis of variance (ANOVA) followed by Tukey’s multiple comparison tests. (*p < 0.05, **p < 0.01; n = 25 to 30 cells from 3 independent experiments) (**c,d**) Network formation analysis in cortical cultures after treatment with F22 at 100 μg/ml for 3 days and under control conditions. Different channels for cell bodies, connections and processed images are represented. Student’s *t*-test, *p < 0.05, **p < 0.01, ***p < 0.001 (n = 80 images for each condition from 4 independent experiments).

Neuronal activity within the CNS depends on ATP availability, which is generated by oxidative metabolism in mitochondria. Thus, mitochondrial metabolic activity can be used as a measure of neuronal function^17^. Here, we treated primary neuronal cultures with a range of nanoliposome concentrations and measured their metabolic activity in order to assess F22 biocompatibility using the Cell Proliferation Kit II (XTT, Sigma-Aldrich) as per manufacturers protocol (Fig. 2, see Supporting Information for details). Figure 2a illustrates the metabolic activity of neural cells treated with various concentrations of F22 at different time points; the same number of cells was used in all experiments. Treatment with F22 lecithin nanoliposomes induced a statistically significant increase in metabolic activity of cells at the highest concentration tested (100 μg/ml) compared to control. The intermediate concentrations and the lowest concentrations only induced significant effects at 7 days *in vitro* (DIV) (Fig. 2a).

To investigate the effect of F22 nanoliposomes on stimulating neurite outgrowth, cortical neurons in culture were treated for three days with F22 at the concentration of 100 μg/ml. A Sholl analysis was performed to quantify neurite outgrowth. The Sholl analysis is a morphometric method, which is widely used in neuroscience to measure the arbor complexity and the neurite architecture in neuronal network^18^. The cultures were stained with the cytoskeletal marker β-III tubulin (refer to Supporting Information for more details). The overall neurite length was longer in neurons treated with F22 nanoliposomes in comparison to the control group. Furthermore, the neurite branching pattern was significantly more complex in the regions more proximal to the cell body compared to the control (Fig. 2b). The data from the Sholl analyses also confirmed the positive effect of F22 in neurite arborization in primary cortical cultures.

Loss of neural connections resulting from accelerated synaptic degeneration and/or death of nerve cells is one of the common symptoms in several neuropathological conditions. The positive effect of F22 in increasing neurite outgrowth could therefore facilitate and promote the maturation of neural networks under pathological conditions. In this regard, we studied the effect of a nanoliposome treatment on the network formation of primary cortical cells. We quantified the connectivity of neuronal networks in two-dimensional cultures based on the statistical density of neurites (see Supporting Information). The cultures were treated with F22 for three days at the same concentration that was used for the Sholl analysis, followed by the same protocol for fixation and staining. The results showed an increase in the network formation rate and its complexity for treated cells compared to the control (Fig. 2c,d). This finding corresponded with data obtained from the Sholl analysis, indicating the positive role of F22 in promoting dendritic and axonal arborization as well as increasing the complexity of the formed neuronal network.

**Figure 3:**
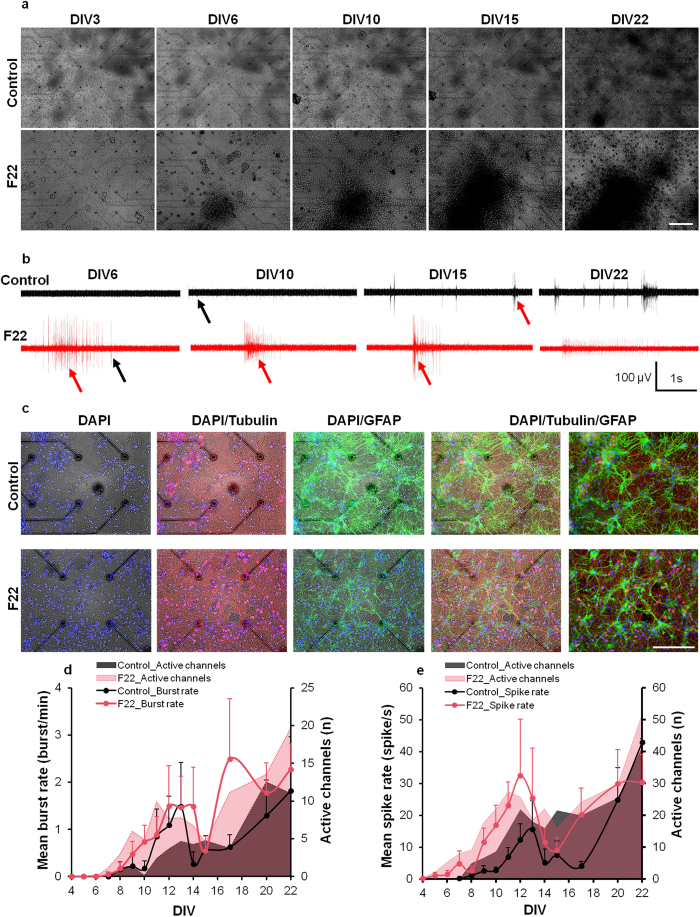
Morphology and activity of cultured networks on MEAs. (**a**) Phase contrast microscopy images from selected controls and F22-treated networks on MEAs from 3 to 22 DIV. For each DIV, a magnified view of the whole network (upper left inset) shows the neurons on and around the four central MEA electrodes (Ø30 μm, 200 μm pitch). (**b**) Raw signals recorded from individual electrodes. Each row represents a five seconds activity window of one electrode at different DIVs. Black arrows point out single spikes and red arrows burst activity. The top row depicts activity recorded from a control culture. The bottom trace was selected from the F22-treated group. F22 clearly shows the appearance of both individual spikes and burst activity at 6 DIV. Spike amplitude ≥100 μV. (**c**) Immunofluorescence images prepared from control and F22-treated cultures at 22 DIV. Each row represents a selected sample from one group. The first four columns show the immunofluorescence images merged with the phase contrast images. Left to right: DAPI, DAPI/ β-tubulin III, DAPI/GFAP, DAPI/β-tubulin III/GFAP (scale bar; 200 μm). (**d**) Average spike frequency and number of active channels. The spike frequency is the mean number of spikes per second recorded from every active electrode in the control or F22-treated cultures, respectively. The mean spike rate was calculated by averaging the mean spike frequency over all active channels in one group. Channels were considered active if they showed ≥3 spikes per minute. Solid lines with circular markers represent the mean ± SEM spike rate, whereas the shaded areas quantify the mean number of active channels in every culture of the control or F22-treated groups on the respective DIV. (**e**) Average burst rate and mean number of electrodes, which showed burst activity. The burst rate is the mean number of bursts per minute recorded on active electrodes in control or F22-treated cultures, respectively. Channels which showed at least 1 burst per minute were considered active channels. Solid lines with circular marks represent average burst rates, whereas the surfaces represent the mean number of active channels in every control or F22-treated cultures on the respective DIV.

To assess the electrophysiological activity of F22-treated neurons, the microelectrode array (MEA) technique was used to determine spontaneous networks activity. Representative microscopy images of networks grown on MEAs are shown in Fig. 3. Concurrent with our previous results, F22-treated cultures formed a denser neuronal network after 6 DIV (Fig. 3a,c).

Electrophysiological activity was evaluated after 4 DIV. In F22-treated cultures, individual spikes were already observed at 5 DIV while their activity onset was delayed to 7 DIV in controls (Fig. 3b). The mean spike rate (spikes/s) per active electrode was considered as an activity index over time and was calculated and compared between the two groups. The mean spike rate per recording electrode in the F22-treated cultures was higher than the control over the entire duration of the study (14.67 ± 6.41 Hz *vs.* 10.44 ± 2.84 Hz). However, the differences were not statistically significant in day to day comparisons (p > 0.05 *vs.* control group at the same DIV; Fig. 3e). The mean number of active channels (mean number of electrodes which showed at least 3 spikes per minute) was also higher in the F22-treated group (18.76 ± 5.36 *vs.* 13.33 ± 5.68; Fig. 3e).

While local spike activity was seen on one or few electrodes, burst activity was defined as episodes of spike flares on many channels, which lasted several milliseconds to seconds^19^. The burst activity in each group was represented as the mean burst rate on each active electrode. The first burst activity was recorded at 6 DIV in the F22-treated group, which was earlier than control (from 8 DIV in one culture). In addition to the overall increase in the mean burst activity per electrode in the F22-treated group (0.92 ± 0.42 bursts/min *vs.* 0.56 ± 0.36 bursts/min), the mean number of active channels which showed at least one burst per minute was higher for F22 compared to control throughout the experiment (6.38 ± 3.1 *vs.* 3.29 ± 2.6; Fig. 3d). Network density and synaptic connections play a crucial role in spontaneous network activity and burst activity^20^,^21^. Increased burst activity and earlier burst onset recorded from F22-treated cultures could be related to the F22-induced increase in neural connectivity and density, which was consistent with our previous results^22^.

**Figure 4:**
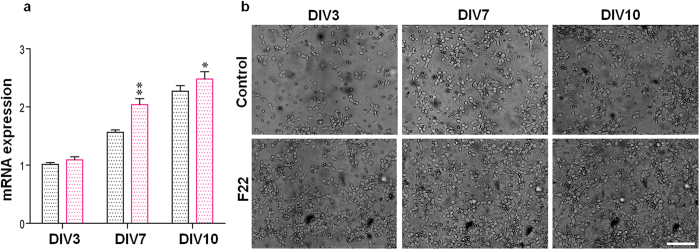
Differential expression of SYN1 in control and F22-treated cultures during neuronal development. (**a**) SYN1 mRNA levels were quantified by qRT-PCR at 3, 7 and 10 DIV. (**b**) Representative phase contrast microscopy images of primary cortical neurons in control and treated samples at various DIVs (scale bar: 50 μm). *p < 0.05; **p < 0.01; Student’s *t*-test was employed for statistical analysis.

In order to find out whether F22 affects synaptic protein expression, we studied synapsin I (SYN1) gene expression as an exemplary protein expression model during neural network development. SYN1 belongs to the family of phosphoproteins, which are involved in synaptogenesis, modulation of neurotransmitter release and nervous system development^23^,^24^. By comparing gene expression in treated and control cultures, we observed an augmentation in SYN1 mRNA levels as a result of F22 administration (Fig. 4). The increase in mRNA levels was observed after 7 and 10 DIV, but not at an earlier developmental stage (3 DIV), which is in agreement with previous studies (Fig. 4b). The data suggests that F22 potentially affects the expression of synaptic proteins, which is in accordance with our results on network formation and activity onset.

## Conclusions

In this work, we developed a biomimetic lecithin (F22) containing various PUFAs. F22 was used to form nanoliposomes, which significantly enhanced the metabolic activity of cortical neurons. Moreover, these nanoliposomes promoted neurite outgrowth and arborization. Consistent with these results, we showed that salmon-derived lecithins facilitated the formation and enhanced the complexity of neuronal networks. MEA recordings from F22-treated cultures also showed an earlier onset of synchronous activity of the cultured neurons. Furthermore, F22 upregulated the expression of SYN1, which is an important factor in synapse development. All these results suggest that F22 affects network development and activity onset by increasing survival rates and enhancing the subsequent formation of network connectivity. These results provide a proof of principle for future studies on the potential of F22 in treating neurodegenerative diseases.

## Experimental Section

### Materials

F22 lecithin derived from natural animal sources was extracted enzymatically without the need of organic solvents. Methanol, boron trifluoride, and hexane were purchased from Sigma–Aldrich (France). Analytical grade solvents were utilized for all the experiments.

## Methods

### Fatty acids composition assessment

Ackman’s protocol was followed for preparing fatty acid esters (FAMEs), which were then analyzed by a Shimadzu 2010 gas chromatograph (Shimadzu, France). The GC was equipped with a flame-ionization detector and a fused silica capillary column (60 cm, 0.2 mm i.d. × 0.25 μm film thickness, P23150 Supelco). The temperature of injector and detector was set to 250 °C, while the column temperature was programmed to remain at 120 °C for 3 min and then rise to 180 °C at the rate of 2 °C/min. The temperature was then increased and maintained at 220 °C for 25 min. Standard mixtures (PUFA1 from marine source; Supelco, Sigma–Aldrich, Bellefonte, PA, USA) were utilized to determine various fatty acids (Table S1).

### Lipid classes

A Iatroscan MK-5 TLC-FID analyzer (Iatron Laboratories Inc., Tokyo, Japan) was utilized to determined lipids within F22. Samples were spotted on Chromarod S-III silica-coated quartz rods, which were then placed in hexane/diethyl ether/formic acid (80:20:0.2, v:v:v) for 20 min. The samples were oven-dried for 1 min at 100 °C and scanned by the Iatroscan analyzer under hydrogen and air flow rates of 160 ml/min and 2 ml/min, respectively. Polar lipid concentration was determined by a migration assay using a polar eluent of chloroform, methanol, and ammoniac (65:35:5). The results were expressed as the average of ten separate samples. The standards employed for identifying the composition were purchased from Sigma (Sigma–Aldrich Chemie GmbH, Germany):

-Neutral lipids: 1-monostearoyl-rac-glycerol, tripalmitin, cholesterol, 1.2-dipalmitoyl-sn-glycerol.

-Phospholipids: L-a-phosphatidylcholine, L-a-phosphatidylinositol, lyso-phosphatidylcholine, 3 sn-phosphatidylethanolamine, L-a-phosphatidyl-L-serine, sphingomyelin.

### Preparation of nanoliposomes

To create the nanoliposomes, a 2% (w/w) solution of F22 was prepared and stirred for 4 h under nitrogen atmosphere. The mixture was then sonicated (SonicatorVibra cell 75115, 500 watt, Bioblock Scientific Co) at 40 kHz and at 40% of full power for 120 s (1 s on and 1 s off). Nanoliposomes were stored in the dark at 4 °C for further use.

### Liposome size measurement by dynamic light scattering (DLS)

A Malvern Zetasizer Nano ZS (Malvern instruments, UK) was used to estimate nanoliposome size distribution. The solutions were mixed with ultra-filtrate distilled water (ratio of 1:400) and were placed in cylindrical cells (10 mm diameter). The scattering intensity was measured at an angle of 173° relative to the source at 25 °C. Intensity autocorrelation functions were analyzed by a general purposed algorithm (integrated by the Malvern Zetasizer software).An average of five different measurements are reported as the size of nanoliposomes.

### Electrophoretic mobility

Electrophoretic mobility (μE) was measured by laser doppler electrophoresis. Samples were placed in a standard capillary electrophoresis cell with integrated gold electrodes. A diluted nanoliposome solution (ratio of 1:400 in DI water) was used for measuring their electrophoretic mobility by the Malvern Zetasizer Nano ZS discussed above. Three different samples were measured and the mean was reported as the μE.

### Nanoparticle tracking analysis (NTA)

NTA is a nanoparticle visualization technique that provides nanoparticle size, count, and concentration, even when the systems are complex and polydisperse (Figure S1). A digital microscope LM10 system (NanoSight, Salisbury, UK) was used for NTA experiments. Diluted samples were injected into the cell using by a syringe and the video images of particle movement under Brownian motion were analyzed to estimate their size distribution. The measurements were done at 25 °C, and video clips were captured over 60 s.

### Analysis of incoherent polarized steady light transport (AIPSLT)

An experimental setup similar to the one described by Dillet *et al*. was employed here (Figure S2-A)^25^. The system includes a light source (100 μm diameter spot light at a wavelength of 635 nm) and a CCD camera (1 cm^2^). Utilizing liquid crystal retarders, the polarization state of the incident and backscattered photons are changed, which allows recording a series of 16 images. The combination of these images is used to form the Mueller matrix (Fig. S2-B)^4^. The Mueller matrices are then used to obtain the transport length and the average particle volume inside the turbid solution. The transport length is calculated using the curve of radial intensity decrease from the barycenter of the M11 image, corresponding to unpolarized light transport. The average particle size is then calculated from the others images of the Mueller matrix, and all normalized by the transport length.

### Transmission electron microscopy (TEM)

TEM was utilized to monitor nanoliposome morphology with a negative staining method. Nanoliposome samples were diluted in distilled water (ratio of 1:10) and were mixed with 2% ammonium molybdate solution (1:1 ratio). The mixture was left for 3 min at room temperature and a drop of this solution was placed on a Formvar carbon-coated copper grid (200 mesh, 3 mm diameter HF 36). After drying, a Philips CM20 TEM equipped with an Olympus TEM CCD camera was used to determine the morphology at 200 kV.

### Atomic force microscopy imaging (AFM)

Images of the different supported F22 bilayers (SLBs) were acquired on a Bruker AFM Dimension FastScan (Bruker, Billerica, MA, USA) with NPG tips (Bruker, Billerica, MA, USA) and spring constant of about 0.32 N/m (constructor data). Images were obtained at room temperature in Peak Force QNM mode, shortly after SLB formation. Images of 5, 10 and 20 μm size were acquired at least for two different samples and two different areas per sample. Images were analyzed by Nanoscope Analysis (v140r2). Depth analyses as well as profile analyses were performed for each image to determine bilayer heights.

### Cortical cell culture

Cortices were dissected from E18.5 embryos in ice-cold PBS, incubated with trypsin (0.125%) for 15 min at 37 °C, and mechanically dissociated. Neurons (50,000 cells/well) were then resuspended in neurobasal medium containing 10% horse serum, 2 mm glutamine and antibiotics (plating medium, all Invitrogen Life Technologies) and plated on poly-D-lysine-coated glass coverslips. After 3 hours, the medium was removed and replaced with neurobasal containing 2% B27 supplement, 2 mm glutamine and antibiotics (maintenance medium, all Invitrogen Life Technologies). For biocompatibility test, neurons were directly plated on 96 wells plates (15,000 cells per well) in neurobasal medium containing 2% B27 supplement, 2 mm glutamine and antibiotics. All procedures were approved by Italian Ministry of Health and in accordance with the guidelines established by the European Community Council.

To evaluate the effect of rapeseed treatment on network activity, cortical neurons were also cultured on microelectrode arrays (MEAs; Multichannel Systems, Reutlingen, Germany) consisting of 60 planar electrodes (10 or 30 μm diameter, 200 μm electrode pitch).

One day before cell seeding, MEA surfaces were hydrophilized by oxygen plasma treatment 0.5–2 min, 60 W, 2.45 GHz, 0.4 mbar O_2_), and coated with 10 μl poly-D-lysine (0.1 μg/μl, Sigma) and laminine (0.05 μg/μl, Sigma) to support cell adhesion. MEA surfaces were rinsed 3 times with sterile ultrapure water for five minutes, and dried in a vacuum chamber. The cell suspension (20 μl) with a total number of 100,000 neurons were added onto each MEA and incubated for 20 minutes (5% CO_2_, 37 °C, 95% RH) to allow the cells to attach to the coated surface. Warmed 1.5 ml serum-free cell culture medium (Neurobasal medium, B27 2%, Glutamax 1%, penicillin/streptomycin 1%) was added to each MEA and cultures were kept in an incubator over the entire study period except for the recording and imaging sessions.

### Quantification of metabolic activity

The metabolic activity of the cortical neurons was assessed using the Cell Proliferation Kit II (XTT, 11 465 015 001, Roche, Mannheim, Germany) as per manufacturers protocol. 6 hours after plating, neurons were treated with nanoliposomes prepared from F22 lecithin at 5 μg/ml, 50 μg/ml and 100 μg/ml (n = 17), or maintained in maintenance medium as non-treated control. Mitochondrial activity was measured at 3, 7 and 10 days *in vitro* (DIV).

### Immunofluorescence assay

Cells were fixed with 4% PFA and 3% sucrose in PBS for 10 min and then permeabilized with 0.1% Triton X-100 for 5 min. Samples were then blocked with 3% BSA and 2% goat serum in PBS for 30 min. Primary and secondary antibodies were also diluted in 3% BSA and 2% goat serum solution and incubated for one hour at room temperature. Hoechst (33342, 2.5 mg/ml, Sigma) was added to stain cell nuclei. The primary antibodies used here include polyclonal anti-neuronal class III β-tubulin (#T2200, Sigma), monoclonal anti-glial fibrillary acidic protein (GFAP) IgG (#G3893, Sigma). Fluorescent-conjugated secondary antibodies were obtained from Molecular Probes (Invitrogen) and used for staining. An upright Leica TCS SP5 AOBS TANDEM confocal microscope was used for image acquisition from regular and MEA cultures. Images were further processed by using ImageJ and Adobe Photoshop CS3 software.

### Morphological analysis of cortical neurons

To analyze the neurite growth and branching, cells were treated with 100 μg/ml F22 for 3 days and were then fixed and stained with anti-β-III tubulin antibodies. Neurites were identified using the NeuronJ plugin of the ImageJ software. The Sholl plugin for ImageJ was used to perform Sholl analysis with the starting radius 10 μm, radius step size 5 μm, and ending radius 500 μm. Details of image analysis of neural network formation is provided in the Supplementary Information.

### Multielectrode array (MEA) spike analysis

Cortical neurons were cultured on MEAs as described above. Network activity was recorded using the commercially available 60-channels MEA filter-amplifier system (0,1 Hz–25 kHz, 1200× amplification, MEA1060-upright-standard, MCS) with an A/D conversion card (64-channels, 25 kHz sampling frequency/channel, PCIbus) and software interface (MC_Rack, MCS). During recordings, the temperature was kept at 37 °C using a built-in thermal sensor and heating element controlled by an external temperature controller (GEFRAN 800, MCS).

Raw signals were filtered and analysed offline. Low frequency noise was filtered by a second-order Bessel high-pass filter (cut-off at 200 Hz) and spikes were detected by passing a negative threshold set to ±4.5 StDev of the peak-to-peak noise. Only downward threshold-crossings were analyzed.

Spike trains were transformed to time stamps (NeuroExplorer, Nex Technologies); mean spike and burst frequencies and the number of electrodes which had recorded activity were analyzed. The following variables were extracted as numerical results and used for further analysis: 1) mean number of active electrodes on each MEA, 2) mean spike frequency (spikes/s/per active electrode) in control or treated groups, 3) mean number of active electrodes on each MEA which showed burst activity, 4) mean burst frequency on active electrodes on each MEA (bursts/min/electrode). Only those channels which recorded at least three spikes per minute were considered as active channel. Bursts were distinguished from single spikes according to the following criteria: maximum interval between spikes and burst start = 0.02 s, maximum interval between spikes and the end of a burst = 0.01 s, minimum interval between bursts = 0.01 s, minimum burst duration = 0.02 s, minimum number of spikes in a burst = 4, and bin size = 1s. The burst rate was represented as the number of bursts per minute on each electrode and channels were considered active if they showed at least one burst per minute.

### RNA Preparation and qRT-PCR

Total cellular RNA was extracted using Trizol (Invitrogen), and isolated RNA was subjected to DNase I (Promega) treatment. cDNA was synthesized starting from 0.5 μg of treated RNA according to the High-Capacity cDNA Reverse Transcription Kit manual (Applied Biosystems) and used for qRT-PCR. Transcripts were amplified using the following primers: Syn1-FW, 5′-AGC TCA ACA AAT CCC AGT CTC T-3′; Syn1-RV, 5′-CGG ATG GTC TCA GCT TTC AC-3′; HPRT1-FW, 5′-TCA GTC AAC GGG GGA CAT AAA-3′; HPRT1-RV, 5′-GGG GCT GTA CTG CTT AAC CAG-3′; GAPDH-FW, 5′-AGG TCG GTG TGA ACG GAT TTG-3′; GAPDH-RV, 5′-TGT AGA CCA TGT AGT TGA GGT CA-3′.

### Statistical analysis

All values were expressed as mean ± S.D. The data obtained from the metabolic activity test were statistically analyzed using one way ANOVA followed by Bonferroni for multiple comparison tests. Statistical significance was determined by paired *t*-tests; *p* < 0.05 was considered to be significant (*p < 0.05, **p < 0.01, ***p < 0.001). The data obtained from the Sholl analysis were analyzed by the Student’s *t*-test.

For the analysis of MEA data, mean spike and burst frequencies were averaged over all active electrodes of each group at each DIV and compared between groups. All data was analyzed using GraphPad Prism (version 5). A two-way variance analysis (ANOVA) compared two groups over the entire recording period between 4 to 22 DIVs. It was followed by the Bonferroni post-test to compare two groups at each time point separately. Data are represented as Mean ± SEM.

## Additional Information

**How to cite this article**: Latifi, S. *et al*. Natural lecithin promotes neural network complexity and activity. *Sci. Rep.* **6**, 25777; doi: 10.1038/srep25777 (2016).

## Supplementary Material

Supplementary Information
