# Supplementary material for: Natural lecithin promotes neural network complexity and activity
Source: Sci Rep. 2016 May 27;6:25777. doi: 10.1038/srep25777 (PMC4882550; doi:10.1038/srep25777)
Supplement: Supplementary Information [file srep25777-s1.doc]

**Natural Lecithin Promotes Neural Network Complexity and Activity**

Shahrzad Latifi, Ali Tamayol, Rouhollah Habibey, Reza Sabzevari, Cyril Kahn, David Geny, Eftekhar Eftekharpour, Nasim Annabi, Axel Blau, Michel Linder, Elmira Arab-Tehrany

Image processing and analysis of neural network formation

To calculate the network density, RGB images were taken. The three color channels were used to decompose images into network connections and cell bodies (nuclei). Let I=[I_R_;I_G_;I_B_] be an image represented as a three-dimensional array, where I_R_, I_G_, and I_B_ are the three channels represented as matrices concatenated along the third dimension of the image array. Because cell compartments were stained with different dyes, cell bodies appeared in green, and network connections in red and could thus be decomposed.

To remove noise caused by the imaging device, the *green* channel was thresholded using a set of hypotheses to extract cell bodies. Afterwards, possible connections between adjacent cell bodies were separated by morphological operators. Among these hypotheses, sufficiently large somata with circular shape were considered as a set of representative neuronal cell bodies.

Assume $\tilde{I}_{C}$ as the initial estimation of a picture that contains only cell bodies. Such initial estimation is obtained by comparing the value of each pixel in the *Blue* channel with a threshold value
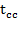
.

,

where i=1… b, indicates the pixel index and represents the value of pixel i in image . Now, consider R- {r_1_, …, r_m_} as a set of
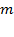
 regions in representing candidates for cell bodies. Such regions will be accepted as cell bodies if they meet the size and shape criteria. Such that:

,

where $\tilde{C}$ is a subset of R in which the area of its elements $c_{j}$ is bigger than a certain threshold . Now, members of set
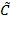
 have to be checked to see whether their shapes look like a circle. So, we have

$C\subset\tilde{C}| Circulatory (c_{k}\in C) > t_{ce}$,

where C is the set of image regions c_k_ identified as cell bodies and t_ce_ is a threshold to evaluate the shape of image regions c_k_ by their similarity to a circular shape. Circularity is defined as the ratio of the distance between the ellipse foci and the major axis of the ellipse, such that

$$Circulatory\equiv\frac{d}{l}$$

where d is the distance between the ellipse foci and l is the length of the ellipse’s major axis. This ratio will be 0 for circular shapes and will be l for a line segment.

| 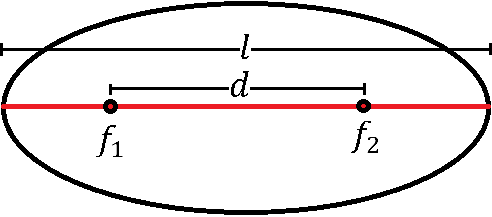 | 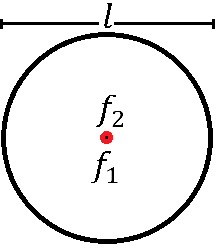 | 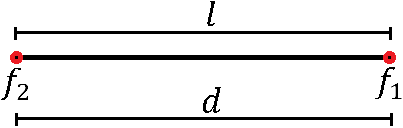 |
| --- | --- | --- |
| $Circulatory\equiv\frac{d}{l}$ | $Circulatory\equiv0$ | $Circulatory\equiv1$ |

Putting together all valid regions c_k_, we have:

$I_{c}\equiv\cup c_{k}$,

where $I_{c}$ is the image that only contains regions c_k_ representing the cell bodies. A binary mask M_c_ is extracted from $I_{c}$ where the value for each pixel i in the mask M_c_ is obtained by:

$$M_{ci}=\left\{\begin{matrix} 0, & I_{ci}=0 \\ 1, & I_{ci}>0 \end{matrix}\right.$$

where $M_{ci}$ indicates the value of mask $M_{c}$ at pixel i.

In order to extract the network connections, a threshold is applied on the *Red* channel to remove the acquisition noise. The resulting image for the *Red* channel is then multiplied by the binary mask $M_{c}$ representing pixels belonging to cell bodies. This step removes the connections surrounding the cell bodies, in order to avoid their effect on the network density value.

$$M_{Ni}=\left\{\begin{matrix} 0, & I_{Ni}=0 \\ 1, & I_{Ni}>0 \end{matrix}\right.$$

To validate the detected cell bodies, those that are surrounded by a patch of connections are considered as valid cell bodies and the others will be eliminated from the set of cell bodies. Similarly, patch of network connections that are not surrounding any cell body will be removed and only valid connections will remain in the network.

To calculate the network density, number of pixels used by the valid network connections is divided by the number of cell bodies in the sample. To eliminate the scale effect resulting from variations in the focal length, the computed network density is normalized by the average area of the cell bodies in the image.

Thus, network density *D* is calculated as

$$D=100\times\frac{\sum_{i=1}^{b} M_{Ni}}{b.\sum_{k=i}^{\left\|C\right\|} Area (c_{k})}$$

where $\left\|C\right\|$ is the number of elements in set C.

**Table S1**: Main fatty acid composition of F22 by gas chromatography (area %).

| Fatty acids | F22 | |
| --- | --- | --- |
| C14:0  C16:0  C18:0  C21:0  C23:0 | % | SD |
|  | 1.21  12.11  4. 11  1.80  1.42 | 0.01  0.03  0.04  0.01  0.01 |
| SFA | 20.65 | - |
| C16:1  C17:1  C18:1n9 | 1.23  1.35  20.18 | 0.06  0.08  0.01 |
| MUFA | 22.76 | - |
| C18:2n6  C18:3n3  C20:4n6  C20:5n3 (EPA)  C22:4n6  C22:5n3  C22:6n3 (DHA) | 5.80  4.68  2.23  9.56  1.22  3.10  30 | 0.01  0.02  0.08  0.06  0.02  0.06  0.29 |
| PUFA | 56.59 | - |
| n-3/n-6 | 5.11 | - |
| DHA/EPA | 3.13 | - |


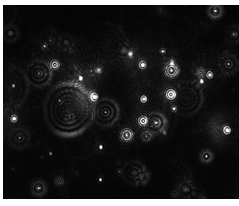


**Figure s1.** NTA video frame demonstrating the fabricated nanoliposomes from F22.


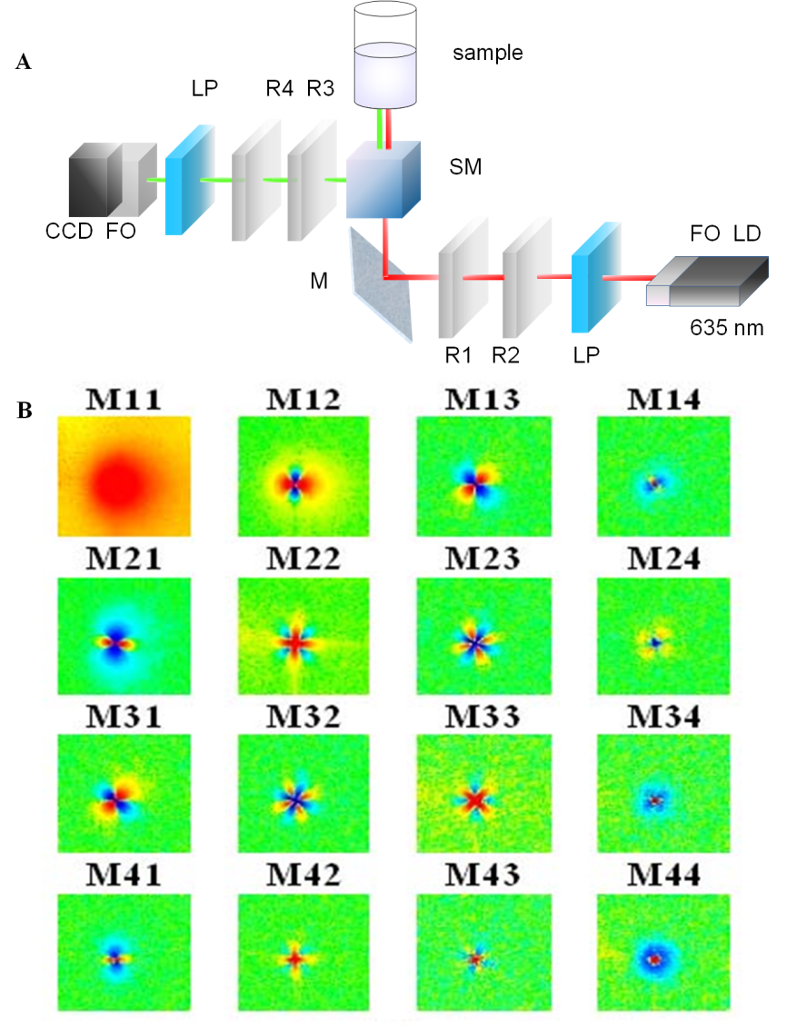


**Figure s2.** AIPSLT experimental scheme and an example of Mueller Matrix, LD: Laser Diode, FO: Focusing Optic, LP: Linear Polarizer, R1,R2,R3 and R4: liquid crystal Retarders, M: polarizer-maintening Mirror, SM: polarizer-maintening Semitransparent Mirror, CCD: Charge-Coupled Device.
